# Palm Mixed-Carotenes Modulate Viability, Wound Closure and Osteoprotegerin mRNA Expression in Human Periodontal Ligament Stem Cells

**DOI:** 10.3390/biomedicines14091897

**Published:** 2026-08-25

**Authors:** Yixin Sun, Sook-Luan Ng, Syed Nabil, Xin-Fang Leong

**Affiliations:** 1 Department of Craniofacial Diagnostics and Biosciences, Faculty of Dentistry, Universiti Kebangsaan Malaysia, Kuala Lumpur 50300, Malaysia; p138506@siswa.ukm.edu.my (Y.S.); ngsookluan@ukm.edu.my (S.-L.N.); 2 Department of Oral and Maxillofacial Surgery, Faculty of Dentistry, Universiti Kebangsaan Malaysia, Kuala Lumpur 50300, Malaysia; syednabil@ukm.edu.my

**Keywords:** bone remodeling, human periodontal ligament stem cells, palm mixed-carotenes, periodontal regeneration, periodontitis

## Abstract

**Background/Objectives**: Periodontitis is characterized by the gradual breakdown of tooth-supporting tissues, including the periodontal ligament and alveolar bone, while current regenerative strategies remain limited. Palm mixed-carotenes (PMC), a natural carotenoid-rich compound with antioxidant and cytoprotective properties, may have potential in periodontal regenerative research. However, its effects on human periodontal ligament stem cells (hPDLSCs) remain underexplored. This study evaluated the effects of PMC on hPDLSC viability, wound closure, and osteogenic- and bone remodeling-related molecular responses. **Methods**: Primary hPDLSCs were isolated, characterized, and treated with PMC at concentrations ranging from 0 to 100 μg/mL. Cell viability was evaluated using the 3-[4,5-dimethylthiazol-2-yl]-2,5-diphenyltetrazolium bromide (MTT) assay, wound closure using a wound-scratch assay, gene expression of osteoprotegerin (*OPG*), osteopontin (*OPN*), and osteocalcin (*OCN*) using real-time polymerase chain reaction (RT-qPCR), and secreted protein levels using enzyme-linked immunosorbent assay (ELISA). **Results**: PMC showed a biphasic viability response, with 6.25 μg/mL producing the most favorable effect by increasing the cell viability to 111.6% relative to the negative control. This concentration was selected, together with 12.5 μg/mL, for subsequent assays. In the wound-scratch assay, 6.25 μg/mL PMC significantly enhanced wound closure at 48 and 72 h, reaching 75.17% at 72 h compared with 52.69% in the negative control. Gene expression analysis showed that 6.25 μg/mL PMC significantly upregulated *OPG* mRNA expression compared with the negative control, 12.5 μg/mL PMC, and positive control groups. *OPN* and *OCN* showed limited responses, and PMC did not significantly increase secreted OPG or OCN protein levels. **Conclusions**: Overall, PMC at 6.25 μg/mL demonstrated the most favorable response in hPDLSCs, suggesting its potential as a bioactive candidate for further periodontal regenerative research.

## 1. Introduction

Periodontitis is a chronic inflammatory condition associated with microbial dysbiosis in addition to a dysregulated host immune response. Approximately 89.6 million incident cases of severe periodontitis were estimated globally in 2021, representing a 76.32% increase in the absolute number of incident cases compared with 1990. The corresponding global age-standardized incidence rate was 1069.4 per 100,000 person-years [1]. As the disease progresses, it damages the periodontal ligament, cementum, alveolar bone, and gingiva [2]. Clinically, this destruction may manifest as gingival inflammation, periodontal pocketing, attachment loss, alveolar bone resorption, tooth mobility, and eventual tooth loss [3].

Beyond its local effects on the tooth-supporting tissues, epidemiological studies have reported associations between periodontitis and diabetes mellitus [4] and hypertension [5], while a recent review highlighted potential links between periodontitis and age-related neurodegenerative disorders, including Alzheimer’s and Parkinson’s diseases [6]. Although conventional periodontal therapy is effective in reducing microbial burden and controlling inflammation, complete restoration of the original periodontal architecture remains challenging once tissue destruction has occurred [7]. Therefore, strategies that support periodontal tissue repair and regeneration remain an important focus in periodontal research [8].

Human periodontal ligament stem cells (hPDLSCs) are highly relevant to periodontal regeneration because of their ability to self-renew, migrate, and differentiate into cementoblast-like, osteoblast-like, and fibroblast-like cells [9,10,11]. These cellular responses are important for the repair and regeneration of tooth-supporting structures, particularly the periodontal ligament and alveolar bone. Osteogenic- and bone remodeling-related markers, including osteocalcin (OCN), osteopontin (OPN), and osteoprotegerin (OPG), are commonly used to evaluate cellular responses associated with mineralization, matrix remodeling, and bone homeostasis. OCN is involved in bone mineralization and calcium homeostasis. OPN participates in cell–matrix interactions and bone remodeling. OPG regulates osteoclastogenesis by binding to receptor activator of nuclear factor kappa-B ligand (RANKL) and limiting RANKL–RANK signaling [12,13,14,15].

Carotenoids comprise naturally occurring yellow-to-red pigment compounds that are widely distributed among plants, algae, and photosynthetic microorganisms [16]. Oil palm fruit (*Elaeis guineensis*) is a rich natural source of carotenoids, particularly α-carotene and β-carotene. Growing evidence suggests that carotenoids play vital roles in regulating oxidative stress, modulating inflammation, and promoting wound repair [17,18,19]. These properties may be relevant to periodontal regeneration, as oxidative stress contributes to periodontal tissue destruction, impaired cellular function, and alveolar bone loss [20,21].

Palm mixed-carotenes (PMC), a carotenoid-rich natural compound derived from oil palm fruit, have shown favorable biological effects in several cellular models, including neural cells, retinal pigment epithelial cells, oral mucosal fibroblasts, and osteoblast-related models [22,23,24,25]. However, the direct effects of PMC on hPDLSCs remain largely unexplored. Therefore, this study aimed to evaluate the effects of PMC on hPDLSC viability, wound closure, and osteogenic- and bone remodeling-related markers, with particular emphasis on OCN, OPN, and OPG. The findings may provide preliminary in vitro evidence on the potential of PMC as a bioactive candidate for further periodontal regenerative research.

## 2. Materials and Methods

### 2.1. Study Design

The in vitro study workflow included isolation, culture, and expansion of primary hPDLSCs; cell characterization; PMC treatment; and assessment of cellular responses relevant to periodontal regeneration. The outcomes assessed were cell viability, wound closure, and osteogenic- and bone remodeling-related molecular responses. Ethical clearance was granted by the Research Ethics Committee of Universiti Kebangsaan Malaysia (approval code: JEP-2024-745).

### 2.2. Tooth Collection, Isolation and Culture of Primary hPDLSCs

Extracted human premolars or molars were collected from six young adult patients, with one tooth obtained from each patient. The patients underwent clinically indicated tooth extraction as part of their orthodontic treatment at the Oral Surgery Clinic, Faculty of Dentistry, Universiti Kebangsaan Malaysia. The extracted teeth were collected for research use only after written informed consent had been obtained from the patients. All human-derived samples were handled in compliance with the approved ethical protocol. The inclusion criteria were intact tooth structure, closed apices, and the absence of trauma, periodontal disease, or apical pathology. Teeth exhibiting severe caries, open apices, periodontal disease, or apical pathology were excluded.

Following extraction, residual blood was removed by rinsing the teeth with sterile saline, after which the teeth were transferred into sterile centrifuge tubes containing phosphate-buffered saline (PBS; Sigma-Aldrich, St. Louis, MO, USA, Catalog No.: P4474) supplemented with 2% antibiotic–antimycotic solution (Gibco™, Thermo Fisher Scientific, Waltham, MA, USA, Catalog No.: 15240062). The teeth were stored in an insulated container with ice packs during transport to the laboratory.

For hPDLSC isolation, periodontal ligament tissue was collected aseptically from the mid-root region of each extracted tooth using a sterile surgical blade. The harvested periodontal ligament tissue was finely sectioned and digested with 0.3% type I collagenase (Worthington Biochemical, Lakewood, NJ, USA, Catalog No.: LS004194) at 37 °C for 10 min with gentle shaking at 260 rpm (KS 4000 ic control, IKA, Staufen, Germany). An equal volume of complete culture medium, consisting of Dulbecco’s Modified Eagle Medium/F-12 (DMEM/F-12; Gibco™, Thermo Fisher Scientific, Waltham, MA, USA, Catalog No.: 11320033) supplemented with 10% fetal bovine serum (FBS; Capricorn Scientific, Ebsdorfergrund-Dreihausen, Germany, Catalog No.: FBS-16A) and 1% antibiotic–antimycotic solution, was added to neutralize the enzymatic digestion. The cell suspension was centrifuged at 600× *g* for 10 min (Sorvall™ ST 16R, Thermo Fisher Scientific, Waltham, MA, USA), after which the supernatant was discarded. The resulting cell pellet was resuspended in fresh complete medium and seeded into culture flasks. The cells were maintained at 37 °C in a humidified incubator with 5% CO_2_ (Heracell™ 150i, Thermo Fisher Scientific, Waltham, MA, USA) [26].

The cultures were replenished with fresh medium every 2–3 days. Upon reaching approximately 80–90% confluence, the adherent cell layer was detached for passaging using 0.125% trypsin-EDTA (Gibco™, Thermo Fisher Scientific, Waltham, MA, USA, Catalog No.: 25200-072). The cell suspension was centrifuged at 600× *g* for 10 min, resuspended in fresh complete medium, and subcultured at a 1:3 ratio. Following expansion to the fifth passage, the hPDLSCs were allocated to functional assays and downstream molecular analyses of osteogenic- and bone remodeling-related markers [27].

The periodontal ligament tissue and resulting hPDLSC culture from each patient were processed, expanded, and analyzed separately. Cells derived from different patients were not pooled at any stage. Each independently established patient-derived hPDLSC culture represented one biological replicate. Cell characterization was performed using cultures derived from three patients (*n* = 3 biological replicates), whereas the 3-[4,5-dimethylthiazol-2-yl]-2,5-diphenyltetrazolium bromide (MTT), wound-closure, real-time polymerase chain reaction (RT-qPCR), and enzyme-linked immunosorbent assay (ELISA) experiments were performed using cultures derived independently from all six patients (*n* = 6 biological replicates for each experiment).

### 2.3. Characterization of hPDLSCs

The characterization procedures were performed using protocols adapted from published stem cell studies, with modifications according to the present hPDLSC experimental design [28]. Primary hPDLSCs were characterized at passage 2 based on gene expression of pluripotency-associated markers, mesenchymal stem cell markers, and osteogenic differentiation potential. For gene expression analysis, total RNA was extracted from 1 × 10^6^ cells using TRIzol reagent (Ambion™, Thermo Fisher Scientific, Waltham, MA, USA, Catalog No.: 15596018) following the manufacturer-provided protocol. Extracted RNA was assessed spectrophotometrically to determine its concentration and purity (NanoDrop™ ND-2000, Thermo Fisher Scientific, Waltham, MA, USA), and 200 ng of total RNA was reverse-transcribed into complementary DNA (cDNA).

The expression of pluripotency-associated markers (*OCT4*, *SOX2*, and *NANOG*), mesenchymal stem cell-associated markers (*CD73*, *CD90*, and *CD105*), hematopoietic-associated markers (*CD34* and *CD45*), and immune/antigen-presenting marker (*HLA-DR*) was analyzed by RT-qPCR [28]. Relative gene expression was determined using the 2^−ΔΔ*C*^_T_ method [29] with glyceraldehyde-3-phosphate dehydrogenase (*GAPDH*) used as the internal reference gene for normalization. The primer sequences adopted from Ahmad Shuhaimi et al. [28] are provided in Table 1.

Osteogenic differentiation potential was assessed qualitatively using Alizarin Red S (Sigma-Aldrich, St. Louis, MO, USA, Catalog No.: A5533) staining after 21 days of culture in osteogenic induction medium (Gibco™, Thermo Fisher Scientific, Waltham, MA, USA, Catalog No.: A10072-01). Cells were fixed with 10% formalin (Sigma-Aldrich, St. Louis, MO, USA, Catalog No.: HT501158) for 30 min, stained with 2% Alizarin Red S solution, and examined under a phase-contrast inverted microscope (IX51, Olympus, Tokyo, Japan). The presence of orange-red calcium-associated deposits was interpreted as evidence of extracellular mineralized matrix formation [30].

### 2.4. Preparation of PMC

Palm mixed-carotenes (PMC) were supplied by ExcelVite Sdn. Bhd (EVTene™ 8%, Chemor, Perak, Malaysia). The commercial preparation contained 67% β-carotene and 32% α-carotene with small amounts of γ-carotene and lycopene. PMC working solutions were prepared following the method described by Meganathan et al. [23]. PMC was dissolved in dimethyl sulfoxide (DMSO; Thermo Fisher Scientific, Waltham, MA, USA, Catalog No.: 036480-K2) to prepare a 100 mg/mL stock solution. The stock solution was subsequently diluted in culture medium to obtain the required working concentrations. The final DMSO concentration varied according to the PMC concentration but did not exceed 0.1% (*v*/*v*).

### 2.5. MTT Assay for Cell Viability

Fifth-passage hPDLSCs were seeded in 96-well plates at 5000 cells/well in 100 µL complete medium and incubated for 24 h to allow cell attachment. The medium was then replaced with serum-free DMEM/F-12 supplemented with 1% antibiotic–antimycotic, 1% L-glutamine (Thermo Fisher Scientific, Waltham, MA, USA, Catalog No.: 25030-081), and 1% vitamin C (Sigma-Aldrich, St. Louis, MO, USA, Catalog No.: A92902), containing PMC at concentrations of 1.56–100 µg/mL. Serum-free medium containing 0.1% DMSO served as the negative control, while medium supplemented with 10% FBS was used as the positive control. Blank wells containing medium without cells were included for background correction.

After 72 h of treatment, 10 µL of MTT working solution (Sigma-Aldrich, St. Louis, MO, USA, Catalog No.: SIG-M5655) was added to each well, and the wells were incubated for 4 h at 37 °C. The supernatant was then removed, and formazan crystals were dissolved in 100 µL DMSO. Absorbance was measured at 570 nm using a microplate reader (Varioskan™ LUX, Thermo Fisher Scientific, Waltham, MA, USA) [31]. Cell viability was calculated after blank correction and expressed as a percentage relative to the negative control [26]. Based on the cell viability results, two selected PMC concentrations were subsequently used for wound closure and molecular analyses.

### 2.6. Wound-Scratch Assay for Wound Closure

The wound-scratch assay was adapted from Azmi et al. [32]. Fifth-passage hPDLSCs were seeded in grid-marked 24-well plates and cultured until a confluent monolayer was obtained. Two parallel linear scratches were created across the monolayer in each well using a sterile micropipette tip. The wells were gently washed with PBS to remove dislodged cells and cellular debris before being replenished with the respective treatment media. Cells were assigned to four groups: negative control, positive control, and two PMC-treated groups selected based on the MTT assay results.

**Figure 1:**
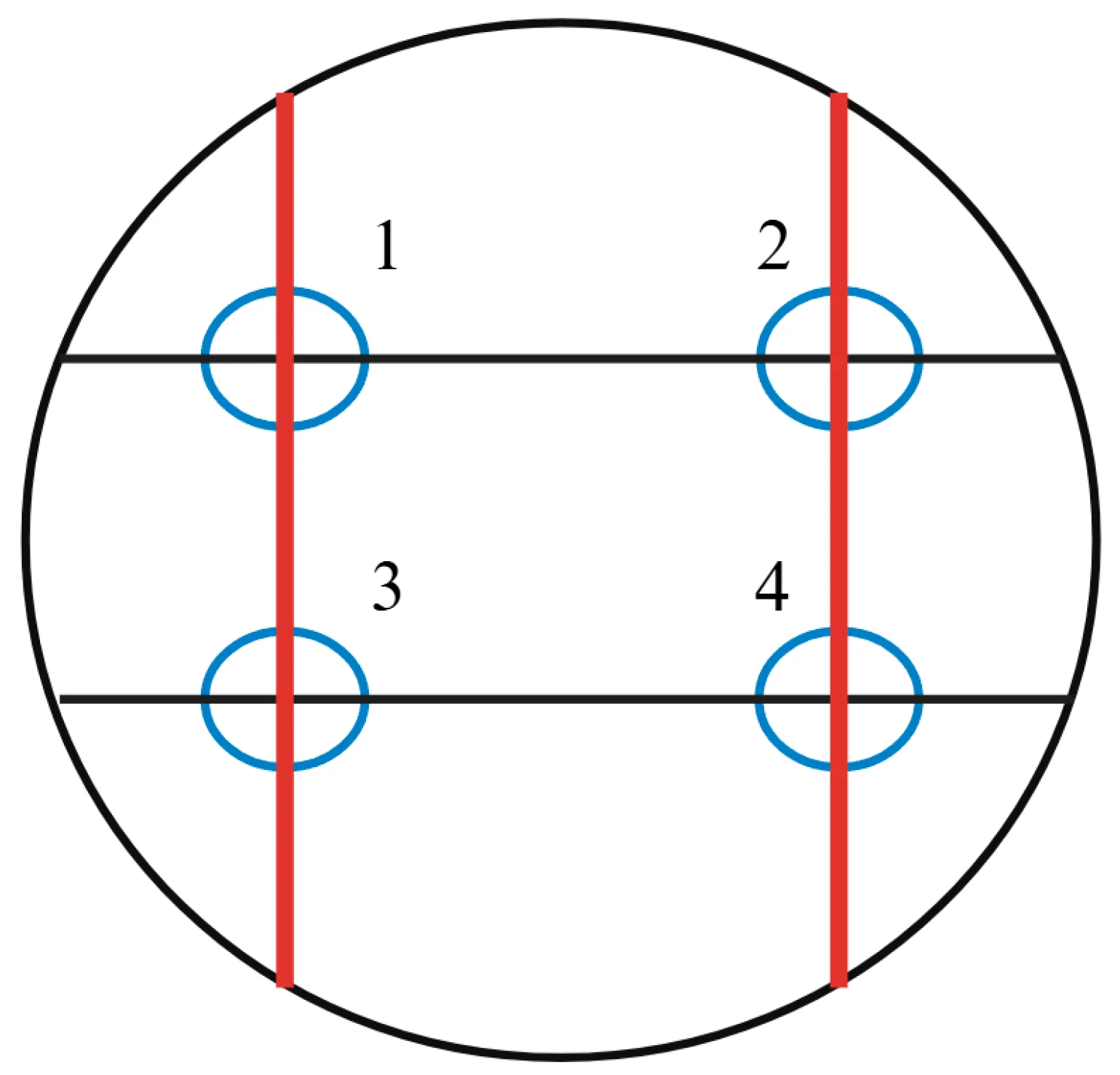
Schematic representation of the four predefined imaging locations used in the wound-scratch assay. The intersections of two parallel scratch lines (red) and two horizontal reference grid lines (black) defined imaging locations 1–4 (blue circles) within each well. Created in BioRender. Leong, X. (2026) https://BioRender.com/9z2fee2, (accessed on 17 August 2026).

Two horizontal reference grid lines were marked on the underside of each well. The intersections of the two scratch lines with the two reference grid lines formed four predefined imaging locations. Images of the same four locations were captured immediately after scratching (0 h) and subsequently at 24, 48, and 72 h using an inverted microscope at 100× magnification. This approach ensured consistent repositioning and imaging of the same fields across all time points (Figure 1).

Wound area was defined as the cell-free area within the scratch and was measured using AxioVision software version 4.8 (Carl Zeiss Microscopy GmbH, Jena, Germany). The contour function was used to delineate the wound margins, and the wound areas measured at the four predefined locations were averaged for each well. Wound-area measurements were performed by an investigator blinded to the treatment allocation. Wound closure was expressed as the percentage reduction in wound area from the baseline measurement at 0 h. At the same time, the wound closure rate was determined from the reduction in wound area over time and expressed as area reduction (μm^2^) per hour. After the final imaging time point, cells and culture supernatants were harvested for subsequent RNA and protein analyses, respectively.

### 2.7. Molecular Analysis for Osteogenic- and Bone Remodeling-Related Markers

#### 2.7.1. Collection of Conditioned Supernatants and Cell Lysates

Following completion of the wound-scratch assay at 72 h, conditioned culture supernatants were collected from each well and stored at −80 °C until ELISA analysis. The adherent cells from the same wells were then gently rinsed with sterile PBS and lysed directly using TRIzol reagent. Cell lysates were collected into RNase-free microcentrifuge tubes and stored at −80 °C until RNA extraction. This approach ensured that secreted protein levels and gene expression analyses were assessed under the same treatment conditions.

#### 2.7.2. RT-qPCR Analysis of OCN, OPN and OPG Gene Expression

Total RNA was extracted from cell lysates using TRIzol reagent according to the manufacturer’s protocol, which involved chloroform-based phase separation, isopropanol precipitation, ethanol washing, and final dissolution of the RNA pellet in RNase- and DNase-free distilled water. The concentration and purity of the extracted RNA were determined using a NanoDrop™ ND-2000 spectrophotometer (Thermo Fisher Scientific, Waltham, MA, USA). Complementary DNA (cDNA) was synthesized from 200 ng of total RNA using qScript™ cDNA Synthesis Kit (QuantaBio, Beverly, MA, USA, Catalog No.: 95047-100). Primer annealing was carried out at 23 °C for 10 min, followed by reverse transcription at 50 °C for 60 min and enzyme inactivation at 85 °C for 5 min.

RT-qPCR was performed using SensiFAST SYBR No-ROX kit (Bioline, London, UK, Catalog No.: BIO-98005) on a thermal cycler system (C1000™, Bio-Rad, Hercules, CA, USA). The thermal cycling conditions consisted of initial denaturation at 95 °C for 3 min, followed by 40 cycles of denaturation at 95 °C for 10 s and annealing/extension at 61 °C for 30 s. Melting curve analysis was performed to confirm amplification specificity. The forward and reverse primers for osteogenic- and bone remodeling-related markers were designed using Primer3 version 4.1.0 (https://primer3.ut.ee/), based on target gene sequences retrieved from the NIH GenBank database. *GAPDH* was used as the housekeeping gene, and relative gene expression of osteocalcin (*OCN*), osteopontin (*OPN*), and osteoprotegerin (*OPG*) was calculated using the 2^−ΔΔ*C*^_T_ method. Primer details are provided in Table 2.

#### 2.7.3. ELISA Quantification of Secreted OCN, OPN and OPG Proteins

The concentrations of secreted OCN (Catalog No.: E-EL-H1343), OPN (Catalog No.: E-EL-H1347), and OPG (Catalog No.: E-EL-H1341) in culture supernatants were measured using the respective human ELISA kits (Elabscience, Houston, TX, USA) based on the manufacturer-provided protocol. Briefly, standards, blanks, and samples were loaded into the designated wells in duplicate and incubated at 37 °C. Sequential incubation with biotinylated detection antibody and horseradish peroxidase conjugate was then followed, and washing steps were performed between reagent additions. Substrate reagent was then added and incubated protected from light, after which stop solution was added. Absorbance was measured at 450 nm using a microplate reader (uQuant, BioTek, Winooski, VT, USA). Protein concentrations were obtained from the respective standard curves.

### 2.8. Statistical Analysis

Data analyses were performed using IBM SPSS Statistics version 33.0 (IBM Corp., Armonk, NY, USA), and figures were prepared using GraphPad Prism version 10 (GraphPad Software, San Diego, CA, USA). Quantitative data are expressed as mean ± standard error of the mean (SEM). Data normality was assessed using the Shapiro–Wilk test. One-way analysis of variance (ANOVA) and Tukey’s post hoc multiple comparisons test were used for cell viability and molecular analyses. Kruskal–Wallis test was used with Dunn–Bonferroni post hoc pairwise comparisons where applicable. For wound closure data involving repeated measurements over time, two-way mixed ANOVA was used to evaluate the effects of treatment group, time, and group × time interaction, followed by Bonferroni-adjusted pairwise comparisons where appropriate. Statistical significance was defined as *p* < 0.05.

## 3. Results

### 3.1. Characterization of hPDLSCs

**Figure 2:**
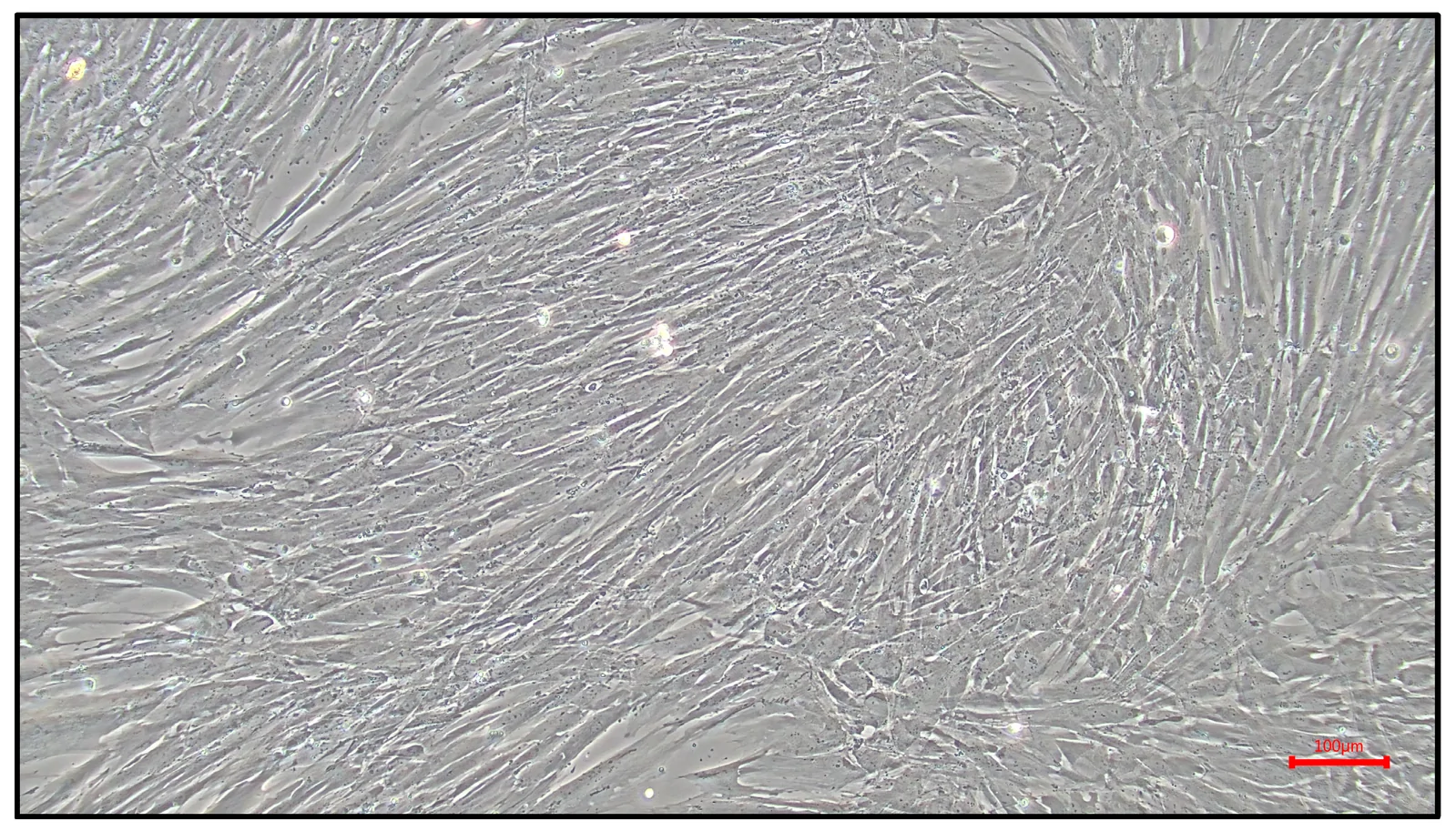
Phase-contrast micrograph showing spindle-shaped, fibroblast-like morphology and whirlpool-like growth pattern of hPDLSCs at Passage 2. Scale bar = 100 μm.

**Figure 3:**
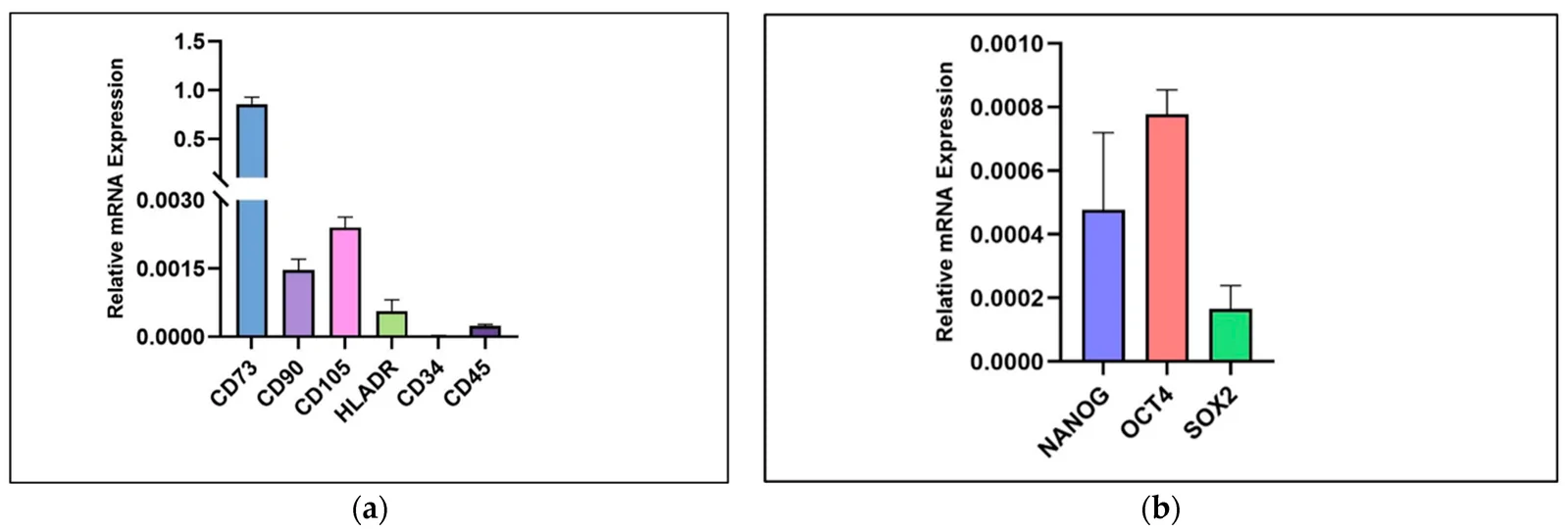
Relative mRNA expression in hPDLSCs determined by RT-qPCR. (**a**) Mesenchymal-associated markers (*CD73*, *CD90*, and *CD105*) and hematopoietic/immunogenic-associated markers (*CD34*, *CD45*, and *HLA-DR*); (**b**) stemness-associated transcription factors (*NANOG*, *OCT4*, and *SOX2*). Data are presented as mean ± SEM (*n* = 3 biological replicates).

**Figure 4:**
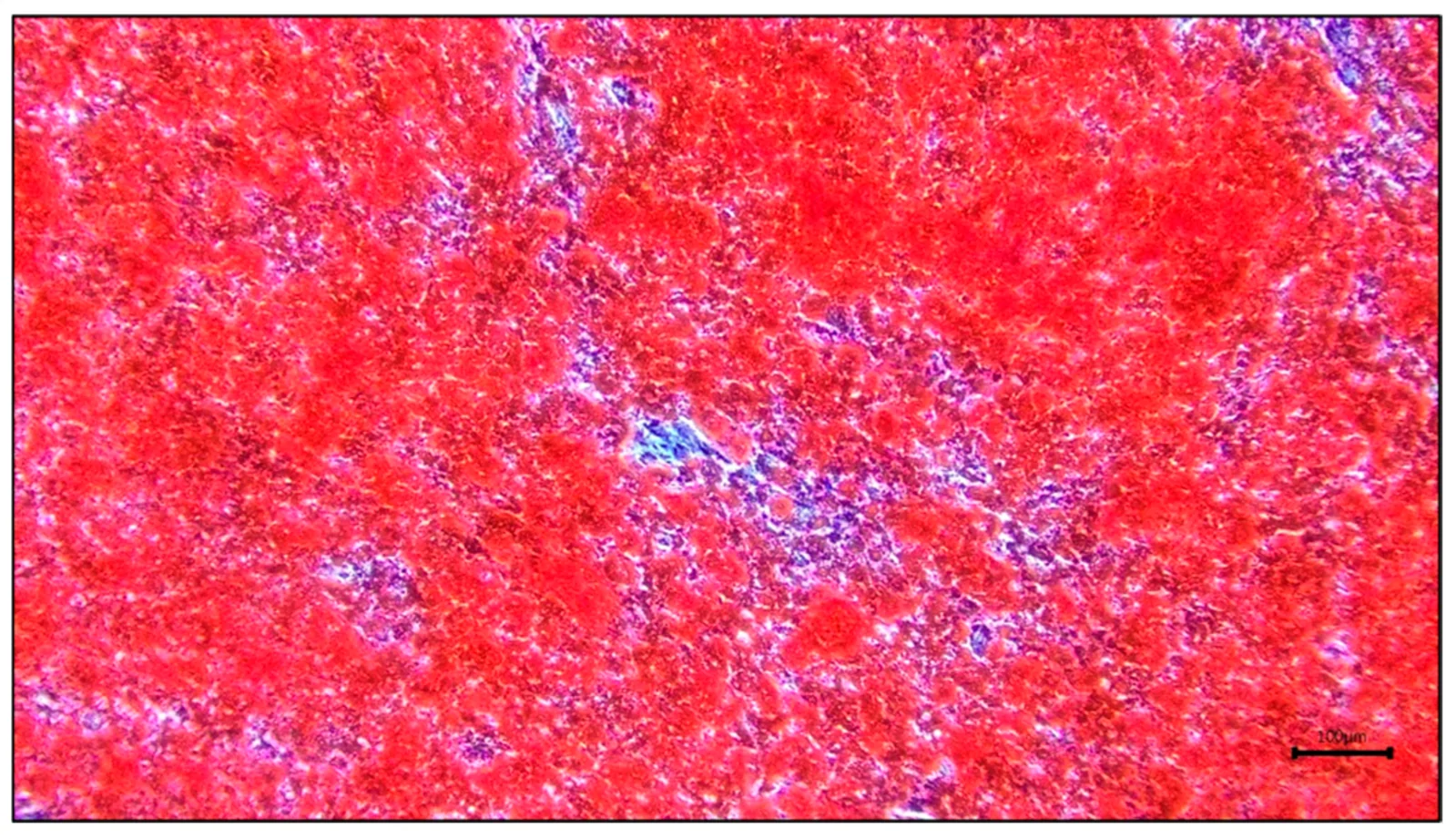
Representative image of Alizarin Red S staining at 100× magnification showing mineralized nodules formed by hPDLSCs following culture in osteogenic induction medium. Red-stained deposits indicate calcium-containing mineralized matrix. Scale bar = 100 μm.

Primary hPDLSCs at passage 2 displayed adherent, spindle-shaped fibroblast-like morphology with a typical whirlpool-like growth pattern, consistent with periodontal ligament-derived mesenchymal stem cell-like cells (Figure 2). RT-qPCR analysis showed detectable expression of mesenchymal-associated markers (*CD73*, *CD90*, and *CD105*), while the expression of hematopoietic/immunogenic-associated markers (*CD34*, *CD45*, and *HLA-DR*) was low or minimal (Figure 3a). Stemness-associated transcription factors *NANOG*, *OCT4*, and *SOX2* were also detected, indicating that the cultured cells retained progenitor-related transcriptional characteristics (Figure 3b). Following osteogenic induction, Alizarin Red S staining demonstrated red calcium-associated mineralized deposits, supporting the osteogenic differentiation potential of the isolated hPDLSCs (Figure 4).

### 3.2. Effects of PMC on hPDLSC Viability

Following 72 h of treatment, hPDLSCs maintained an adherent, spindle-shaped, fibroblast-like morphology across the negative control, 6.25 µg/mL PMC, 12.5 µg/mL PMC, and positive control groups. No obvious morphological features of cytotoxicity, such as marked cell rounding, shrinkage, or widespread detachment, were observed in the PMC-treated groups.

**Figure 5:**
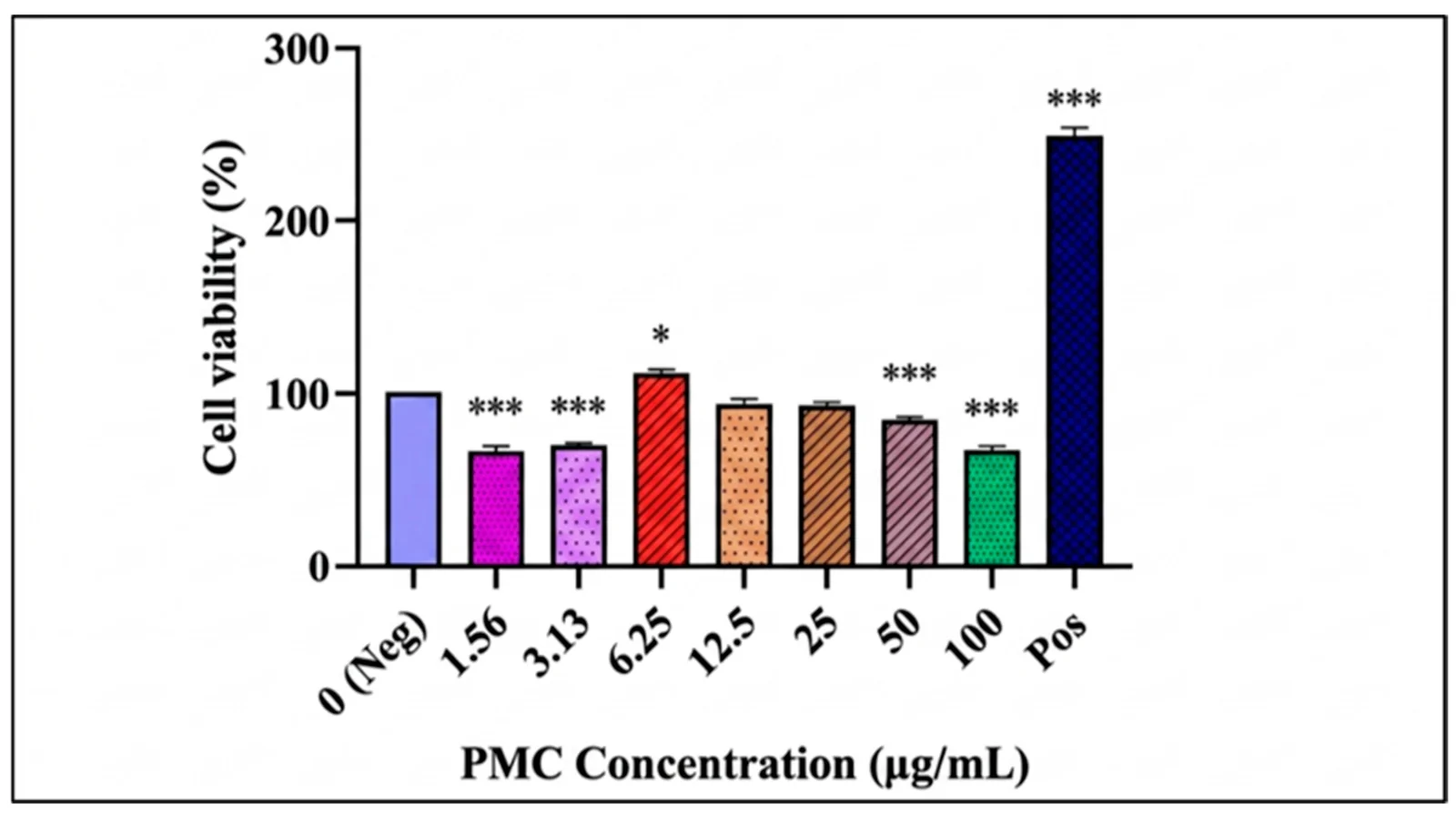
Cell viability of hPDLSCs following 72 h of PMC treatment, expressed as a percentage relative to the negative control (0 μg/mL PMC). Data are presented as mean ± SEM (*n* = 6 biological replicates). Statistical analysis was performed using one-way ANOVA followed by Tukey’s post hoc multiple comparisons test. * *p* < 0.05, *** *p* < 0.001 vs. 0 μg/mL.

Cell viability was assessed using the MTT assay and expressed as a percentage relative to the negative control (Figure 5). PMC showed a biphasic effect on hPDLSC viability. Treatment with 6.25 µg/mL PMC significantly increased cell viability compared with the negative control (*p* = 0.019). In contrast, lower concentrations of 1.56 and 3.13 µg/mL, as well as higher concentrations of 50 and 100 µg/mL, significantly reduced cell viability (*p* < 0.001). No significant differences were observed at 12.5 and 25 µg/mL compared with the negative control. The positive control showed the highest viability response. Based on these findings, 6.25 and 12.5 µg/mL PMC were selected for subsequent wound closure and molecular analyses.

### 3.3. Effects of PMC on hPDLSC Wound Closure

**Figure 6:**
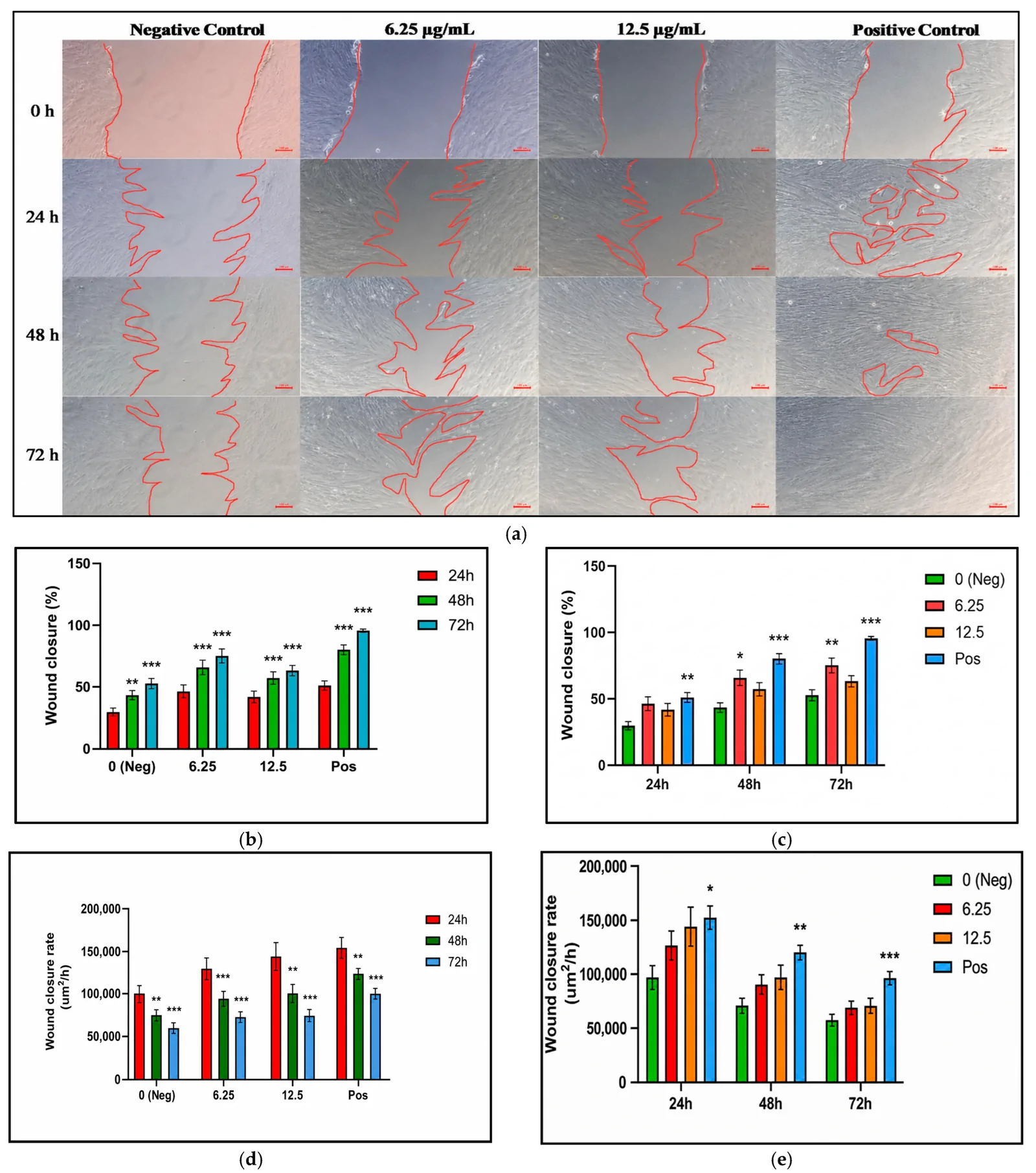
(**a**) Representative images at 100× magnification from the wound-scratch assay showing hPDLSCs wound closure at 0 h, 24 h, 48 h, and 72 h under different treatment conditions: Negative control (0 μg/mL), 6.25 μg/mL, 12.5 μg/mL PMC, and positive control. Scale bar = 100 μm. (**b**) Temporal changes in wound closure (%) within each treatment group at 24, 48, and 72 h. (**c**) Comparison of wound closure (%) among treatment groups at each time point. (**d**) Temporal changes in wound closure rate within each treatment group at 24, 48, and 72 h. (**e**) Comparison of wound closure rate among treatment groups at each time point. Data are presented as mean ± SEM (*n* = 6 biological replicates). Statistical analysis was performed using two-way ANOVA followed by Bonferroni-adjusted pairwise comparisons. In panels (**b**,**d**), asterisks indicate significant differences compared with 24 h within the same treatment group. In panels (**c**,**e**), asterisks indicate significant differences compared with the negative control at the corresponding time point. * *p* < 0.05, ** *p* < 0.01, *** *p* < 0.001.

The wound-scratch assay was performed to evaluate the effects of selected PMC concentrations, 6.25 and 12.5 µg/mL, on hPDLSC wound closure. Representative images showed a clear scratch area at 0 h, followed by progressive wound narrowing over 24, 48, and 72 h in all groups (Figure 6a). Compared with the negative control, the 6.25 µg/mL PMC group showed greater wound reduction over time, particularly at 48 and 72 h. The 12.5 µg/mL PMC group showed moderate wound closure, while the positive control demonstrated the greatest reduction in wound area.

Quantitative analysis showed that wound closure increased progressively from 24 to 72 h within all treatment groups (Figure 6b). Significant increases in wound closure were observed at later time points compared with 24 h in the negative control, 6.25 µg/mL PMC, 12.5 µg/mL PMC, and positive control groups. When wound closure was compared among treatment groups at each time point, 6.25 µg/mL PMC significantly enhanced wound closure compared with the negative control at 48 h (*p* = 0.012) and 72 h (*p* = 0.004), but not at 24 h (Figure 6c). In contrast, 12.5 µg/mL PMC did not significantly increase wound closure compared with the negative control at any time point. The positive control showed significantly higher wound closure than the negative control throughout the observation period (*p* < 0.01).

For wound closure rate analysis, the highest values were observed at 24 h, followed by a progressive decline at 48 and 72 h within all treatment groups (Figure 6d). Significant reductions were observed at later time points compared with 24 h in the negative control, 6.25 µg/mL PMC, 12.5 µg/mL PMC, and positive control groups (*p* < 0.01). When wound closure rates were compared among treatment groups at each time point, the positive control showed significantly higher values than the negative control at 24, 48, and 72 h (Figure 6e). Although both PMC-treated groups showed numerically higher wound closure rates than the negative control, these differences were not statistically significant at any time point.

### 3.4. Effects of PMC on Osteogenic- and Bone Remodeling-Related Gene Expression

**Figure 7:**
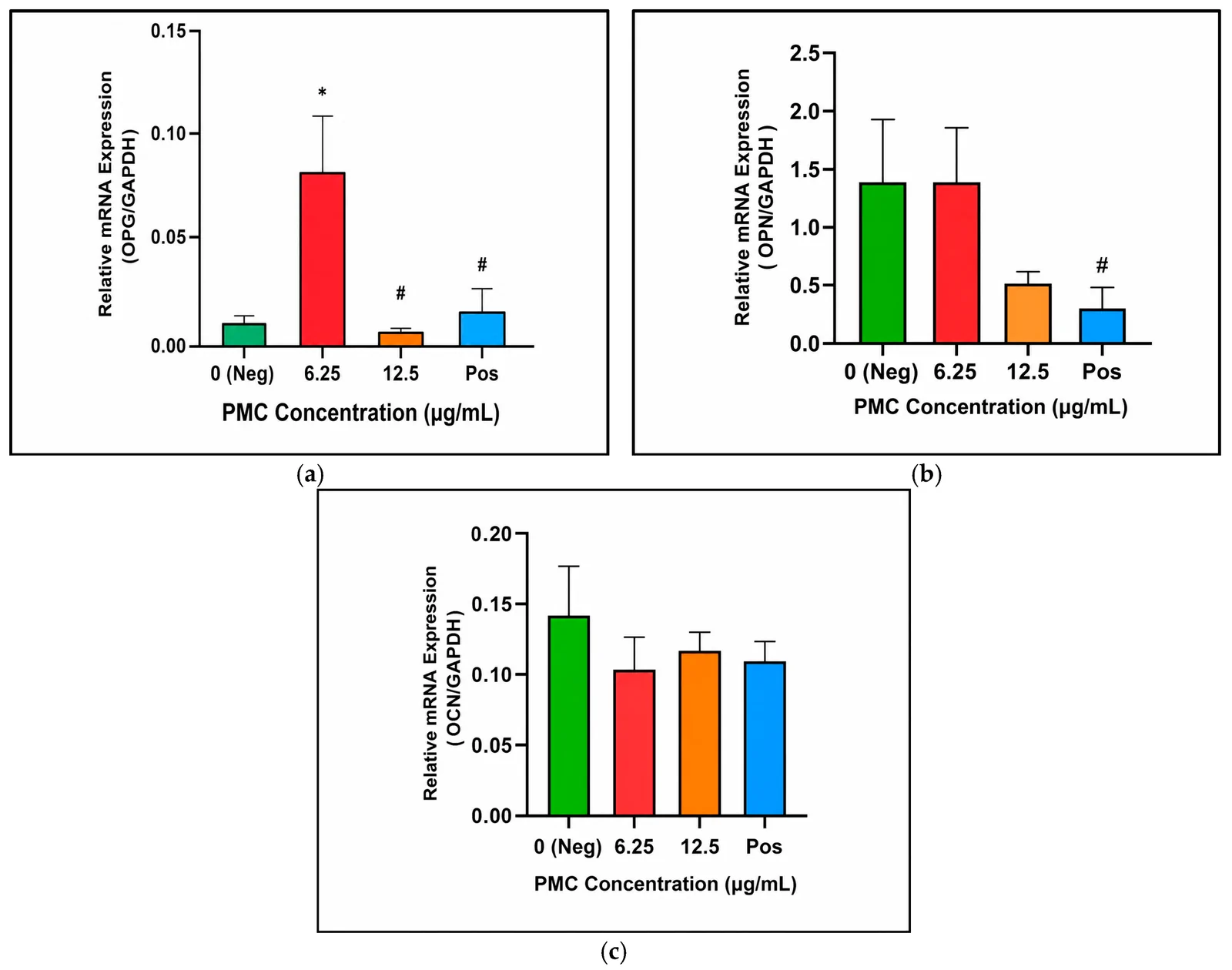
Relative mRNA expression of (**a**) *OPG*, (**b**) *OPN*, and (**c**) *OCN* in hPDLSCs following PMC treatment, as determined by RT-qPCR. Gene expression levels are presented as mean ± SEM (*n* = 6, biological replicates). Statistical analysis was performed using one-way ANOVA followed by Tukey’s post hoc test for *OPG*, and the Kruskal–Wallis test with pairwise comparisons for *OPN* and *OCN*. * *p* < 0.05 vs. 0 μg/mL, ^#^ *p* < 0.05 vs. 6.25 μg/mL.

The effects of PMC on the mRNA expression of osteogenic- and bone-remodeling-related markers were assessed by RT-qPCR on cell lysates collected at the experimental endpoint. Among the markers analyzed, *OPG* showed the most prominent response to PMC treatment (Figure 7a). Treatment with 6.25 µg/mL PMC significantly increased *OPG* expression compared with the negative control (*p* = 0.014), 12.5 µg/mL PMC (*p* = 0.008), and positive control groups (*p* = 0.022). In contrast, 12.5 µg/mL PMC did not increase *OPG* expression compared with the negative control.

For *OPN*, no significant difference was observed between the PMC-treated groups and the negative control (Figure 7b). However, the 6.25 µg/mL PMC group showed significantly higher *OPN* mRNA expression than the positive control group (*p* = 0.026). *OCN* expression showed no significant differences among the treatment groups (Figure 7c).

### 3.5. Effects of PMC on Secreted Osteogenic- and Bone Remodeling-Related Protein Levels

**Figure 8:**
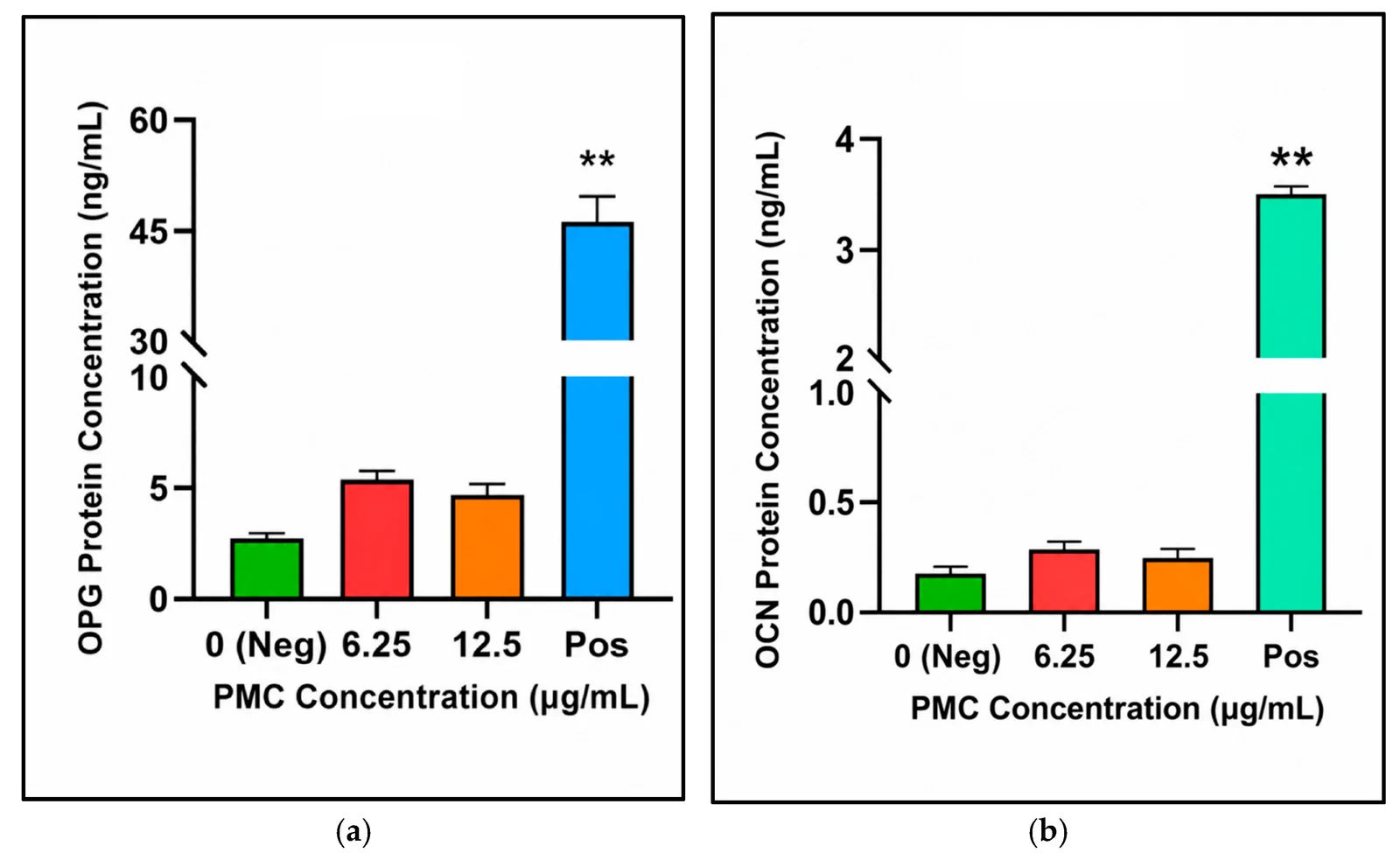
(**a**) OPG concentration and (**b**) OCN concentration measured by ELISA. Data are presented as mean ± SEM (*n* = 6, biological replicates). Statistical significance is indicated by asterisks. ** *p* < 0.05 vs. 0, 6.25, or 12.5 μg/mL.

The concentrations of secreted osteogenic- and bone remodeling-related proteins in hPDLSC culture supernatants were quantified by ELISA. For OPG, the positive control group showed significantly higher protein levels than the negative control and both PMC-treated groups (*p* < 0.001) (Figure 8a). Although the 6.25 and 12.5 µg/mL PMC groups showed higher mean OPG levels than the negative control, these differences were not statistically significant.

For OCN, the positive control group showed significantly higher protein levels than the negative control (*p* < 0.003) and the 12.5 µg/mL PMC group (*p* = 0.016) (Figure 8b). No significant difference was observed between the PMC-treated groups and the negative control. OPN protein levels were below the assay quantifiable range and were therefore excluded from quantitative group comparison.

## 4. Discussion

The present study examined the response of primary hPDLSCs to PMC exposure across functional and molecular endpoints relevant to periodontal regeneration. The isolated cells demonstrated typical hPDLSC characteristics, including spindle-shaped fibroblast-like morphology, expression of mesenchymal-associated markers, low or minimal expression of hematopoietic- and immune-associated markers, detectable stemness-associated transcription factors, and osteogenic differentiation potential. These findings support the suitability of the cell population for subsequent evaluation of PMC-mediated responses. Similar approaches that combine morphological assessment, marker expression analysis, and osteogenic differentiation have been used to support hPDLSC identity before downstream functional assays [33,34,35,36].

PMC produced a biphasic effect on hPDLSC viability. Among the PMC concentrations tested, 6.25 µg/mL significantly increased cell viability, whereas lower and higher concentrations did not yield a similarly favorable response. This suggests that the effect of PMC was not concentration-dependent in a linear manner but occurred within a limited concentration window. The observed findings are in agreement with published studies demonstrating that PMC may exert favorable viability-related effects in selected cell types and exposure ranges, including neural cells, retinal pigment epithelial cells, oral mucosal fibroblasts and osteoblast-related models [22,23,24,25].

Based on the current viability findings, 6.25 µg/mL was selected as the most favorable concentration. Although both 12.5 and 25 µg/mL did not significantly reduce cell viability, 12.5 µg/mL produced the next highest mean viability after 6.25 µg/mL, whereas a lower mean response was observed at 25 µg/mL. As there was no significant difference between 12.5 and 25 µg/mL, the lower concentration was selected as the higher comparison concentration to minimize PMC exposure while retaining a favorable viability response. This selection enabled subsequent wound closure and molecular analyses to be conducted using concentrations that were well tolerated, as assessed by cell morphology and viability.

The positive control produced the strongest response in several assays, particularly cell viability, wound closure, and secreted protein levels. This is expected because the positive control medium supplemented with fetal bovine serum contains a complex mixture of growth factors, attachment factors, proteins, hormones, and nutrients that collectively support cell survival, proliferation, migration, and protein synthesis [37,38]. In contrast, PMC is a defined carotenoid-rich compound and may not be able to reproduce the full biological support provided by serum-containing medium. Therefore, the comparison with the positive control should be interpreted as a reference for favorable cellular responsiveness rather than as a direct equivalent to PMC treatment.

The wound closure findings further support the favorable response observed at 6.25 µg/mL. In the wound-scratch assay, 6.25 µg/mL PMC significantly enhanced wound closure at later time points compared with the negative control, while 12.5 µg/mL showed a less pronounced effect. This wound closure response may be relevant to periodontal repair, as the migration and proliferation of periodontal ligament cells may support cellular repopulation during tissue regeneration [39]. Similar scratch-based assays have been used in hPDLSC studies to evaluate treatment-related effects on wound closure and migratory behavior [40,41,42]. Overall, these findings indicate that 6.25 µg/mL PMC supported wound closure behavior more effectively than 12.5 µg/mL under the present experimental conditions.

At the molecular level, OPG appeared to be the most responsive marker following PMC treatment in the current study. The significant upregulation of *OPG* mRNA at 6.25 µg/mL suggests that PMC may modulate a bone remodeling-related marker in hPDLSCs. This finding is biologically relevant because OPG is involved in the RANKL/OPG regulatory system, which contributes to the control of osteoclastogenesis. Recent work has also mapped cells expressing *RANKL* and *OPG* mRNA in bone tissue, highlighting their roles in the cellular regulation of osteoclastogenic activation [43]. In contrast, *OPN* and *OCN* showed limited transcriptional responses in the present study. This selective transcriptional response suggests that PMC may preferentially affect *OPG* as a bone remodeling-related marker, rather than producing a broad osteogenic marker response across *OPN* and *OCN*.

The ELISA findings showed that PMC treatment did not significantly increase secreted OPG or OCN protein levels compared with the negative control, although the 6.25 µg/mL group showed higher mean values. The discrepancy between *OPG* mRNA expression and secreted protein levels may reflect differences between transcriptional activity and final measurable protein abundance. Protein levels are influenced by multiple regulatory processes beyond mRNA expression, including translational efficiency, secretion kinetics, extracellular matrix retention, degradation, and protein stability [44,45,46,47]. Therefore, the molecular findings should be interpreted within the context of the present experimental design.

When compared with previous studies on antioxidant bioactives in periodontal-related cells, the present findings show both similarities and important differences. Astaxanthin, a xanthophyll carotenoid, has been reported to support periodontal ligament fibroblast migration, collagen production, and osteogenic differentiation, suggesting that carotenoid-based antioxidants may influence cellular behaviors relevant to periodontal repair [48]. Similarly, quercetin, a flavonoid antioxidant, has been shown to protect hPDLSCs against oxidative-stress-induced injury through nuclear factor erythroid 2-related factor 2-related antioxidant signaling and preservation of osteogenic potential [49]. In human gingival fibroblasts, quercetin also enhanced wound-healing responses while reducing oxidative stress, senescence-associated changes, inflammatory cytokine production, and nuclear factor kappa-B signaling [50]. These studies support the broader concept that antioxidant compounds may enhance cellular resilience and repair-associated responses in periodontal-related cell models.

However, the experimental context of the present study differs from many previous antioxidant-based studies. Several studies evaluated antioxidant compounds under stress- or pathology-associated conditions, such as oxidative stress, advanced glycation end product stimulation, high-glucose exposure, or lipopolysaccharide (LPS)-induced inflammatory stimulation [48,49,50,51]. In contrast, the present study was conducted in hPDLSCs under non-induced basal culture conditions. This distinction is important because stress-induced models may provide a greater opportunity for antioxidant compounds to demonstrate protective or restorative effects. Therefore, the favorable effects of PMC on hPDLSC viability and wound closure observed here may reflect modulation of baseline hPDLSC behavior rather than reversal of experimentally induced cellular injury. Although these effects may be partly related to the antioxidant bioactivity of PMC, oxidative-stress markers and antioxidant signaling pathways were not directly examined in the present study.

Differences in experimental duration may also explain the limited OPN and OCN responses observed in the present study. In an LPS-stimulated human periodontal ligament cell model, quercetin was reported to increase *OPN* and *OCN* mRNA expression after treatment for up to 14 days [51]. In contrast, the present study evaluated gene expression at 72 h under non-induced basal culture conditions. Since OPN and OCN are associated with osteogenic differentiation and matrix mineralization over longer culture periods, the 72 h endpoint may have been too early to detect a broad osteogenic transcriptional response. This may explain why PMC selectively increased *OPG* mRNA expression without producing marked changes in *OPN* or *OCN*.

Several limitations should be considered. First, this study was conducted under controlled in vitro conditions, which cannot fully replicate the complex periodontal microenvironment involving multiple cell types, inflammatory mediators, extracellular matrix interactions, microbial factors, and vascular components. Second, the MTT assay reflects viability-associated metabolic activity and does not distinguish between increased cell number and enhanced metabolic activity per cell. Third, the wound-scratch assay reflects collective wound closure and cannot fully separate migration from proliferation. In addition, molecular analysis was limited to selected osteogenic- and bone remodeling-related markers at a single endpoint, while ELISA quantified only secreted proteins in the culture supernatant. The ELISA measurements were not normalized to endpoint cell number or total cellular protein; therefore, differences in cell abundance may have influenced the measured protein concentrations. The final DMSO concentration was not matched across the experimental and control groups. Although the DMSO concentration did not exceed 0.1%, differences in vehicle exposure may have contributed to the observed responses and limit their attribution exclusively to PMC.

Future studies should employ vehicle-matched controls across all treatment groups and incorporate complementary proliferation assays, normalized protein measurements, time-course analyses, broader osteogenic and bone remodeling marker profiling, inflammatory or oxidative stress models, RANKL/OPG assessment, mechanistic pathway evaluation, and more complex experimental systems, such as co-culture, three-dimensional scaffold-based models, and in vivo validation, to better define the biological relevance and translational potential of PMC in periodontal regenerative research.

## 5. Conclusions

To the best of our knowledge, this is the first study to evaluate the effects of PMC on hPDLSCs. The findings suggest that PMC modulates selected cellular and molecular responses relevant to periodontal regeneration in hPDLSCs. Among the concentrations tested, 6.25 µg/mL PMC showed the most favorable overall response by enhancing cell viability, promoting wound closure, and upregulating *OPG* mRNA expression. However, the increase in *OPG* mRNA expression was not accompanied by a significant increase in secreted OPG protein concentration. Collectively, these findings constitute preliminary in vitro evidence and should not be interpreted as confirmation of regenerative activity within the periodontium.

## Figures and Tables

**Table 1:** Primer sequences used for characterization of hPDLSCs.

Marker Type	Gene	Primer Sequence (5′–3′)	Amplicon Length (bp)	GenBank Accession Number
Housekeeping	*GAPDH*	F: CAATGACCCCTTCATTGACC R: TTGATTTTGGAGGGATCTCG	160	NM_002046.5
Pluripotency	*NANOG*	F: TCCTCCTGCGTGAGTCTCTC R: ATACAGGGGCAGGGTCAGAG	199	NM_024865
	*OCT4*	F: GCAAAGCAGAAACCTCCTGTG R: AACCACACTCGGACCACCATC	172	NM_002701
	*SOX2*	F: ATGGGTTCCGGTGGTCAAGT R: ACATGTGAAGTCTGCGCGTC	166	NM_003106
Mesenchymal stem cells	*CD73*	F: CCAGCAGTTGAAAGTGGTGC R: CTGTCAACAAAGCCAGTCTCT	196	NM_002526
	*CD90*	F: TGGGTGAAAGAGCAGGCCT R: CACACAGTGGCCTCATTTC	122	NM_006288
	*CD105*	F: CCTACGTTGCTGGTCTCTATC R: CGAAAGGTAGCCACATGGTG	174	NM_000118
Immuogenic and hematopoietic	*HLA-DR*	F: GTCAATGTTCACGTGTGTCG R: TCCACCCTCAGTGCTTAAAC	149	NM_019111.5
	*CD34*	F: CTCAGCTCAATGCCCTCATT R: AGCCACCCTTCACCTTCTTG	191	NM_001025109
	*CD45*	F: ATGATTGCTGCTGACCGTGG R: TCTCCCCAGTCACTGAGCACA	140	NM_002838

**Table 2:** Primer sequences of housekeeping gene, osteogenic- and bone-remodeling-related markers for RT-qPCR analysis.

Target Gene	Primer Sequences (5′–3′)	Amplicon Length (bp)	GenBank Accession Number
*GAPDH*	F: CAATGACCCCTTCATTGACC R: TTGATTTTGGAGGGATCTCG	160	NM_002046.5
*OCN*	F: GGCAGCGAGGTAGTGAAGAG R CAGCCAACTCGTCACAGTCC	130	NM_1991783.6
*OPN*	F: ATCTCCTAGCCCCACAGACC R: CAATGGAGTCCTGGCTGTCC	111	NM_000582.3
*OPG*	F: CTATACTGCAGCCCCGTGTG R: GGGGTTCCAGCTTGCACC	167	NM_002546.4

## Data Availability

The original contributions presented in this study are included in the article. Further inquiries can be directed to the corresponding author.
